# Then and Now

**Published:** 1896-08

**Authors:** J. A. Robinson


### Then and Now.

BY J. A. ROBINSON, D.D.S.

A paper read at the Annual Meeting of the Michigan State Dental Society, at Grand Rapids, June 12, 1896.

The world acknowledges dentistry as a profession, and profession is a calling or an art coupled with a desire to do something for the elevation of humanity. Like almost every other improvement in our civilization, it grew out of a necessity. The great heart of humanity responded to the silent call of its brothers and sisters in distress or trouble, and it reached out its benevolent hand to aid them and lift them up. And as the world grew into communities, those who gave the most of their time to aid those who were most afflicted were called doctors, and ministers and lawyers were called doctors, and finally it was given to dentists (who were originally doctors) who attended to troublesome teeth, because they were engaged in a labor of love.

### A LABOR OF LOVE.

I.

How did we come by the thoughts of to-day?
They follow along the thoughts that we have,
And follow us through a life's fitful journey,
And never forsake us until we both leave.

II.

All psychical truth is Divine Love and Wisdom ;
It is warp and the woof of the clothing of mind ;
It is what the great Teacher told His disciples :
"If you search with your hearts you will certainly find."

III.

If we could go back to the dawn of Existence,
And see the first life as it came from a pool ;
And see it run on through the Æons of Ages,
We might then, perhaps, account for the whole.

IV.

The objective life remains for the reason,
And Reason is more intensified Light,
And more intense Light is objective contact
That lifts us above the Darkness of Night !

V.

We get all our thoughts from a light correspondence
To things that we know and quite understand ;
Then enlargement of vision and little reflection
Will lift us till we the whole thing can command.

VI.

Harmonious concert of subjective forces ,
In their mission toward us in this world below,
Wide opens the door to the Heavenly Vision
And teaches us all that we really know.

VII.

Then what was the need of Divine Revelation
Except to move on what the inner life feels,
And also confirms, with every pulsation,
The holy religion the inner life yields ?

VIII.

The first human soul found a Holy Alliance
With a Father in Heaven, without Adam's fall ;
And that will continue a lasting affiance,
Till in Heaven we fondly shall follow His call.

IX.

There is in this life a sublime continuity
  Between father and child in this earthly plain,
And when we pass over it never is severed,
  But only the stronger united again.

X.

In Nature the process is always perfected,
  And it can not be true that in this *highest art*,
The Infinite Father would have it subverted,
  And from Infinite processes He would depart.

XI.

When Saul met the Saviour when He had arisen
  And said to him, "Saul, why persecute me?"
It was not to announce a new declaration,
  But to show to the world that others might see!

XII.

Then let us move on till this life is ended
  And the gates are thrown wide to the regions above;
And then we shall know why the Saviour descended,
  And learn that the whole was a mission of Love!

XIII.

The mission of Love gives us Life and its progress
  From small insignificant things that we see,
And hurries us on from our present attainments
In the progress of Life, so the world will be free!

XIV.

All hope is a growth of our former existence
  That we are unconscious but can not forget;
Unseen and unknown, it is part of *gestation*,
  In the fullness of time we shall hear from yet.

XV.

Life itself has no ending, it had no beginning,
  'Tis a part of the Great Life that filleth the world;
'Tis an emblem of Nature growing unto perfection,
  And growing in beauty as it is unfurled.

We are standing on a segment of the ceaseless round of Time. Nature and all organic matter is one ceaseless round, and civilization and art, and culture and inventions that make us what we are (and dentistry), are fit emblems of the progress of all things in Nature, and even Nature herself, from the first star-dust of which this planet is composed, to the beautiful world on which we live, with its lovely forests and mountains and rivers, made by Nature herself and improved and beautified by the hand of man as he progressed in modern civilization and culture. Prof. Taft once paid me the *high compliment* of being "the oldest dentist in the world," and as Nature repeats itself year after year, and old people are prone to repeat themselves, and tell how things were when they began life, and what took place in their youth, and recapitulate the experiences of their young life, I thought it might be of interest to some of the younger members of the profession to learn what dentistry was when I began—in the childhood of the profession. It was in the year 1830, or sixty-six years ago. I do not recollect the exact date, but I was an apprentice in a small watch-factory or watch-shop where they repaired watches and made a few new ones every year.

There was a Dr. Dewan (a French dentist), who came to Old Concord, Massachusetts, to start a new business—taking care of the teeth.

There were no dental instruments for sale,,and my master (Mr. Joshua Haynes) set me to work to make some small tools for Dr. Dewan, under his direction, as I was handy in making small things out of steel and other metals that the common blacksmiths could not do—and so I began by making dental instruments.

The first representation of a set of artificial teeth was in the mouth of a Dr. Baker, an old man who called himself a dentist, who came to our house in Old Concord, Mass., to make an artificial set of teeth for my mother; they were not intended for service, but for *show*—they were made of sheep's or calves' teeth sewed on to a piece of leather, to be worn between the lips and gums, and were taken out while eating. They were worn like glass-eyes—to hide a deformity. This was when I was ten or twelve years old. When I worked for Dr. Dewan I was seventeen or eighteen years old. Dr. Dewan came a second time to Concord to work, and brought some human teeth that were said to be taken from the mouths of the soldiers who were killed at the battle of Waterloo, in Napoleon's battles, and were brought to Boston for sale.

I watched Dr. Dewan while he was making his partial sets of teeth out of bone, and assisted him, as I was with him while he giving me the ideas of what he wanted to use his instruments for, so when he returned I made a few small pieces that were worn comfortably; they were tied in with a silk thread that kept them in place by being tied to the teeth that remained in the mouth. I do not think that until 1831 or 1832 there had ever been any artificial teeth made on plates of gold or silver. The first financial crisis began in this country in the year 1828. It soon took possession of all New England, and all business was broken up, and the factories were very soon closed and the towns seemed almost deserted till 1835 or 1836. I was keeping a watch-shop in Lowell, at No. 16 Merrimac Street. There was a Dr. George Mansfield whose office was over my store; he was a medical doctor, but did some work on the teeth. In the dull times I spent a good deal of time in his office, and finally I went into his office and stayed and studied with him a full year, and gave him two hundred and fifty dollars to teach me what he could about the teeth, then went into dentistry.

I then bought me a horse and wagon, and a few instruments and made some, and started out into the country and practiced in the towns where I could find anything to do. My dental office was the bar-room, and my dental-chair I made by seating my patient in a common chair, and placing my foot in another chair, and standing on one foot and resting the head of my patient on my knee of the other leg, and did all my operating in that position. If I operated in a home I placed a low cricket on the floor to sit on, and placed my patient on the floor to sit down with the head resting against my left arm to steady it, and worked with my right hand and arm. Sometimes the girls and boys complained of the awkward position, and then I set a rockingchair across a shoe-box to steady the rockers, and did the work in thatway if I had a long job. I worked in that way more than a year. Drs. Harwwood and Tucker had the best and most extensive business in dentistry in Boston. Dr. Mansfield knew them as they were all physicians; in fact, every dentist was an M.D. Dr. Mansfield introduced me to them, and told them what I could do in gold and silver work and making solders, and as the *bone sections* were out of use and mineral teeth were being used, I was welcomed into their office in Boston.

The Boston dentists were very secret about all their business, and "*No Admittance*" was placed over the door of every dental office—unless you wanted something done to your teeth. The Stockton teeth were introduced, and artificial work was made on gold and silver. A large number of dentists made their own teeth. Dr. N. C. Keep made the most natural in color, and most of the dentists went into tooth-making. I made teeth for quite a number of dentists from casts of the mouth, sent to me at Old Salem, where I resided before I came West. Dr. N. C. Keep succeeded in getting the most natural in color, and I kept a man to manufacture teeth for some dentists near Old Salem.

Dentistry can truly be said to be an American institution. And it came by the art as a *necessity*—for Americans have the poorest teeth among all peoples. We have a bilious climate, and the working people indulge in more luxuries in living than the average nationality in the history of man; and a very large number of improvements have been the product of the American mind. A person does not usually deserve any credit for improvements brought about in mechanical appliances or art, or what we call genius, any more than he does for the year or the place where he was born. When I said in the beginning of this paper that "we were standing on a *segment* of the ceaseless round of Time," I did not wish to be understood as a part of the yearly journey of the earth around the sun, but of the race from barbarism till to-day. The round of Time, from the discovery of America by Columbus to the Chicago Exposition was 400 years, and the round of Time from the Mosaic account of Creation is 7,000 years; and the round of Time from the Arayan life on this planet (according to Mr. John Fiske) is 45,000,000 years, since they have left a history that can be understood. From the Pilgrims till to-day is about 250 years.

This is true of every age that we have passed over, and will be true so long as we have a law of progress. There is no wealth but what we get from the earth, from which we live, and the children we have and educate to be good men and women. Benjamin Franklin discovered the law of Electricity, and Edison has improved the product of his brain that had silently been improving for two hundred years.

The American dentist has taken the lead as an operator of the dental art. Dr. Miller, of Berlin, who has done so much in literature to help the profession, is an American by birth, and only resides in Berlin because he could be more of a helper by a residence in a foreign country than he could by remaining in his native land. The Yankee elements of character have been so dependent on their own resources for gaining a living, being cast on the rocky coast of New England, had become very strong and while they made many mistakes in theology by clinging to the prejudices of past generations they became very strong and suggestive as a whole, and being dependent on themselves, on the rocky coast of New England, to fight their way with the elements as best they could, they became inventive in every department of life, and their strong characters gave them an individuality of inventive genius, so that if we look up the records of the Patent-Office we will find a large share of the patents come through the American mind.

Then, the eastern portion of the continent being settled first gave them their institutions of learning at an early day, then the education and culture they had received led them to try and preserve what they had been endowed with, and the conservative life they led warned them to look out for their physical natures, and finding that the fillings and artificial substitutes were not as good as their natural teeth, these same persons then introduced rubber-work and crowns and bridge-work—and dentistry grew into a profession. I do not know as I ever heard of the nationality of the person who introduced crowns and bridge-work, but I presume he was of Yankee stock.

Neither do I think the riddle more than half solved by the profession that will make artificial dentures perfect, neither will it be till we have a cement that will seal the cavities in the teeth that are decayed and be strong enough to do good solid service in mastication, and a generation born who will learn to take better care of the teeth. I suppose I might add, in passing, that there is in use in this city to-day what is known as a *patent filling* that I have seen every day for the past two years, besides making a good many by the same process, within the past seven or eight years, that will make as great improvement as the rubber and metal work over the bone sections in former years, or crown and bridge-work over old-fashioned methods. I am certain if you will try it you will be as pleased as you have ever been with any new thing you have ever tried. Do you ask me in what way it has improved upon the mallet for filling teeth?

I answer: by reducing the time employed in the operation *one-third;* by abandoning largely the use of the *rubber-dam;* by saving more teeth than can be saved by the mallet on account of *weak walls*, and by making it possible to do it for less expense to the dentist on account of *actual cost*, and in making it just as good, if not better, when finished, for the person who has the work done, and the immense comfort to the patient during the operation. I have daily witnessed the working of this new method for the past two years, and have seen a register of more that *two thousand fillings* that have stood the ordinary work in mastication, from day to day, for the past eight years, and can assure you, gentlemen, it is a *pronounced success*, full as much as good dentistry done by good operators. *Go and try it!*

But first learn how to do it. It has turned Whittier's poetry into prophesy when he says:

> "Step by step since time began
> We see the steady gain of man."

